# Creativity Style and Achievements: An Investigation on the Role of Emotional Competence, Individual Differences, and Psychometric Intelligence

**DOI:** 10.3389/fpsyg.2018.01826

**Published:** 2018-10-10

**Authors:** Raffaella Nori, Stefania Signore, Paola Bonifacci

**Affiliations:** Department of Psychology, University of Bologna, Bologna, Italy

**Keywords:** creativity, age factors, intelligence, individual difference, emotional competence

## Abstract

Psychometric and emotional intelligence are considered as two separate theoretical constructs, although each one has been found to correlate to a certain degree with measures of creativity. The aim of the present study was to analyze whether individual differences such as age and gender, together with psychometric intelligence and emotional competence (EC) predicted creativity. We selected a sample of 376 participants aged 12–88 (mean age = 30.28 years, *SD* = 19.09 years; 224 females) to evaluate relationships between these constructs across lifespan. Participants were administered the Kaufman Brief Intelligence Test-2, the Short Profile of EC, the Creativity Style Questionnaire Revised (CSQ-R) and the Creative Achievement Questionnaire (CAQ). *T*-test on gender differences evidenced that males had higher creativity achievements compared to females. A path analysis was applied to examine the relationships between the CAQ and CSQ-R scores as dependent variables and the potential predictors assessed. Results showed that CSQ-R was significantly predicted by interpersonal emotional competence and marginally by educational level (*p* = 0.058) and intrapersonal emotional competence (*p* = 0.051). On the other hand, the CAQ score was significantly predicted by gender, age, and composite IQ. Discussion is focused on possible theoretical implications.

## Introduction

A long tradition of research suggests that creativity is related to general cognitive abilities (e.g., Guilford, 1967; Kaufman and Plucker, 2011) and increasing evidence underlines a link between creativity and emotional intelligence (EI) (e.g., Guastello et al., 2004; Sánchez-Ruiz et al., 2011). Psychometric and EI are considered as two separate theoretical constructs (Mayer et al., 1999) although each one has been found to correlate to a certain degree with measures of creativity. Whereas some studies investigated dyadic relationships amongst these three competencies (i.e., creativity, psychometric intelligence, and EI), lack of evidence is available as regards how these constructs are related to one another. The present study aims to investigate the relation between psychometric intelligence, EI and creativity, taking into account demographic variables, such as gender and age. In the following sections, we will briefly define these constructs considering their evolution over the life-course and analyze previous studies which investigated relations amongst them.

### Creativity, Psychometric Intelligence, Emotional Intelligence, and Demographical Variables

It is beyond the scope of the present study to summarize the huge amount of studies that tried to define these three constructs, but all such attempts evidenced that creativity, psychometric intelligence and emotional competence (EC) cannot be reduced to single factors and need to be considered as multidimensional competencies.

Creativity is thought to derive from the interaction between the individual’s attitudes, cognitive processes and environment and this interaction produces something both novel and useful within a given social context (Plucker et al., 2004). The creativity literature has seen convergence on some core issues. For example, a basic definition of creativity has pointed out the capacity to produce ideas and products that are both original and useful or task appropriate (Plucker et al., 2004; Kaufman, 2016). However, because of the complexity of the construct, creativity has been studied at different conceptual levels (Guilford, 1967; Rhodes, 1987; Simonton, 2000; Kaufman and Beghetto, 2009), thus considering creative achievement, creativity style, and creative potential. Creative achievement is the actual realization of this potential and refers to the visible results reached by individual in the course of life (Carson et al., 2005). For the assessment of creative achievement self-report measures are generally used (e.g., Creative Achievement Questionnaire – CAQ; Carson et al., 2005) investigating the level of creativity in relation to different domains.

Both classic (i.e., Feist, 1998) and more recent works (Kaufman et al., 2016) have shown that not only cognitive characteristics, but also personality traits predict creative potential and achievement. One such an example is the openness trait, which is one of the most consistent personality predictors of creativity (e.g., Silvia et al., 2014; Conner and Silvia, 2015; Karwowski and Lebuda, 2015; Forthmann et al., 2018). Moreover, researchers have recently begun to study “how” or “in what way” people exhibit their creativity (Houtz et al., 2010), that is their creativity style. Creative style refers to beliefs (e.g., unconscious beliefs, inspiration, and insight) and to the use of particular strategies (e.g., brainstorming, taking a walk, and taking notes) that accompanies creative work. Creativity style can be measured through the Creativity Style Questionnaire (Kumar and Holman, 1989) or its revised form (QSC-R, Kumar et al., 1997).

Finally, a number of studies focused on divergent thinking (DT), as candidate predictor of creative potential. DT is generally measured with tests such as the Torrance Tests of Creative Thinking, (Torrance et al., 1966) that require to produce different responses for each specific item, with the aim to assess fluency, flexibility, originality and elaboration of ideas. However, there are many arguments (e.g., Weisberg, 2006) suggesting that DT tests should not be considered measures of creative potential but rather “estimates of the potential for creative problem solving. DT is not synonymous with creativity” (Runco and Acar, 2012, p. 72). Creative potential, usually defined as the human’s ability to do something new and useful (Sternberg and Lubart, 1999), is more than DT alone (Runco, 1991; Baer, 1993). Indeed, it also involves deductive and inductive thinking (Dunbar, 1997; Vartanian et al., 2003; Weisberg, 2006) and, as outlined in the work of Getzels and Csikszentmihalyi (1976) and reinforced in the later literature referring to the nature of creative thought, the ability to use specific problem solving strategies (Finke et al., 1992; Runco, 1994) and to generate novel and appropriate solutions and outcomes. The present study did not included measures of DT but specifically focused on creative style and creative achievement measures.

The relationship between creativity and intelligence and the role played by intelligence in creativity is one such continuing research question: theoretical mechanisms of the links between intelligence and creativity are still up for debate, specifically the nature of this relationship. Nowadays, the most accredited taxonomy of intelligence is that proposed by CHC theory (Cattell–Horn–Carroll, Alfonso et al., 2005), a three stratum hierarchical model which includes a general (g) factor and a set of broad abilities which, in turn, include a subset of narrow abilities. Psychometric intelligence can be considered a relatively stable trait across the human lifespan (Deary et al., 2010), although a decline in intelligence scores in elderly populations has been reported (Miller et al., 2009; Wisdom et al., 2012), associated with reduced cognitive control (Craik and Byrd, 1982; Manard et al., 2014), and impoverished working memory functioning (Sander et al., 2012). Considering this point of view, creative ability is conceptualized as a part of intelligence. Although some studies have focused on fluid intelligence (e.g., Batey et al., 2010), recent investigations have followed the CHC model’s placement of creativity, which is in fluid intelligence, or long-term storage and retrieval (Kaufman, 2015). Fluency (i.e., the ability to quickly recall a large number of things) has been found (Unsworth et al., 2010) to be primarily accounted for by working memory capacity (which is part of fluid intelligence) and by vocabulary knowledge (crystallized intelligence); its connection with DT, that is creativity, is certainly straightforward (Kaufman et al., 2011; Silvia et al., 2013).

The vast majority of past studies focus on creativity and changing across lifespan in relation to intelligence. Specifically, it has been found that creativity increases from childhood to adulthood (Smith and Carlsson, 1983, 1985), and more specifically when considering creativity tasks involving real-world problems (Wu et al., 2005). Indeed, the age differentiation hypothesis (Anastasi, 1958; for a review see Blum and Holling, 2017) proposes that the structure of cognitive ability varies across respondent age. A prominent theory about the structural development of cognitive abilities claims dedifferentiation in old age (Baltes et al., 1999). According to this dedifferentiation hypothesis, the g-factor explains more variance with increasing age. Moreover, the dynamic differentiation theory states that the development of cognitive abilities in old age is mainly influenced by common sources, resulting in higher correlations between different cognitive abilities. In contrast, the non-dynamic dedifferentiation theory states that changes in different cognitive abilities are due to a common developmental cause which influences all cognitive abilities with an invariant strength with increasing age (for a review see Hartung et al., 2018). We focused on the relation between intelligence and creativity across lifespan. Considering the elderly, Zhang and Niu (2013) found a decline in creativity, while Ueno et al. (2015) found that, in elderly subjects, higher individual creativity, but not IQ scores, were significantly related with EEG complexity of resting state, which is thought to reflect neural network characteristics. In a similar vein, Jung et al. (2010) found an inverse relationship between cortical thickness and the creativity measures, as measured by the CAQ (Carson et al., 2005), thus suggesting that the network was not limited to a specific brain region, nor to the “more is better” notion (Jung et al., 2005) that is often invoked in explaining life-course trajectories. Considering the accepted progressive thinning of cortical areas in the elderly (Fjell et al., 2009), the results presented by Jung et al. (2005) and Ueno et al. (2015) would allow to hypothesize that creativity does not necessarily decrease according to cognitive decline.

Another issue considered by researchers is the creative potential in relation to demographic variables such as gender differences. There are many studies on gender differences, some using very different methodologies, techniques, and populations (for a review see Baer and Kaufman, 2008). Research on the existence and/or direction of gender differences in creativity has produced mixed and inconsistent findings. Some researchers find men as more creative (e.g., Schmidt and Sinor, 1986), whereas others find higher scores in women (e.g., Amabile, 1983; Richardson, 1986). Others still find no gender differences at all (e.g., Dudek and Verreault, 1989; Paguio and Hollett, 1991). These results could be explained by taking into account the type of creative tasks or questionnaire people have to solve or fill in (Hardy and Gibson, 2015). Considering this aspect, men appear more creative when using measures of creative accomplishment (Simonton, 1994; Piirto, 2004), whereas women appear more creative when using measures of creative potential. Collectively, these findings suggest that gender differences may exist at the measurement level for creativity. Different theories have been developed to explain gender differences in creativity. For example, Abra and Valentine-French (1991) suggested that “creative achievement depends on both biological and environmental factors... [and] because men and women differ in both factors, either or both could have produced the achievement difference” (p. 235). Biological theories of gender differences in creativity are theories that examine the theory that androgynous males and females may be more creative than their less androgynous counterparts. As an example, one currently popular explanation is proposed by Pinker and Spelke (2005). These authors suggest that when mean levels are identical on a given trait, men and women often have different normal curves, with men’s curves often being flatter. “[E]ven in cases where the mean for women and the mean for men are the same, the fact that men are more variable implies that the proportion of men would be higher at one tail, and also higher at the other. As it’s sometimes summarized: more prodigies, more idiots” (Pinker and Spelke, 2005, para. 24). Other theories interpreted gender differences considering differences in development in relation to task demand. Hutt and Bhavnani (1976) explained these differences by the fact that preschool girls, who are more linguistically and socially competent than preschool boys, may engage in more symbolic and therefore covert role-play than boys, and that this kind of imaginative activity would not be very obvious to an observer. It should be noted that the behavioral differences observed by Hutt and Bhavnani (1976) are consistent with gender stereotypes. More recently, Baer and Kaufman (2008) proposed a new theoretical framework: the APT model of creativity, which is a hierarchical model that considers different levels of explanation to interpret gender differences. The first level (*initial requirements*) includes things that are necessary (but not sufficient) for any type of creative production; the second (*general thematic areas*) is referred to those skills, traits, and knowledge that promote creativity; the third level is characterized by *domains* where there are limited factors that promote creativity; and finally *microdomains*, each with its own very specialized knowledge that one must master to make creative contributions, such as biology.

The authors suggested that there seems to be some general factor at work that is limiting female accomplishment: the primary general factor being the Initial Requirement of environment. The environments in which male creators work are generally more conducive to creative accomplishment than those of female creators, allowing men to express their creative abilities more regularly than women. These differences can also be found in the opportunities available to male and female children and adults, and differences in the kinds of experiences women and men are likely to have.

Finally, with reference to EI, this can be defined as the ability to perceive, recognize and label emotions accurately, to use emotions to ameliorate reasoning, and to regulate one’s own emotions (Mayer and Salovey, 1997). Many efforts have been devoted to possibly consider EI as a standard intelligence (McCann et al., 2014) and also in these domains hierarchical models have been proposed. The most influential is the four factors model (Mayer et al., 2002) which includes accurate perception and expression, facilitation (use of emotion to aid problem solving), understanding (of the relation between emotions and contexts), and management (regulation of one’s own and others’ emotions). Although EI is related to psychometric intelligence, it is not included in the CHC model and many evidences suggest that it is a separate construct (e.g., McCann et al., 2014). Some studies have recently tested models in which established factors of emotional processing were regressed into factors representing interindividual variance in cognitive functioning (Mathersul et al., 2008; Adolphs, 2009; Wilhelm et al., 2010) with mixed results. A study by Hildebrandt et al. (2015) addressed this issue by means of a latent variable modeling on selected measures of cognition, emotion recognition and emotion perception. These authors found limited uniqueness in emotion expression perception and evidenced that most variance in this ability can be accounted for by face identity processing and general cognitive skills (figural reasoning, working memory, and immediate and delayed memory).

The effects of aging on EC have been studied by means of self-reported questionnaires. For example, in a recent study (Cabello et al., 2014), a lower level of EI was reported in older people compared to younger people in all dimensions of the Mayer-Salovey-Caruso Emotional Intelligence Test (MSCEIT, Mayer et al., 2002) (perceiving, facilitating, and understanding emotions) except for the managing emotions subscale. They also found that EI was significantly correlated with educational level, and that this variable predicts several dimensions of EI over and above the effects of gender and age. These results seem to be in line with neurophysiological evaluations of emotion recognition. For example, a recent meta-analysis suggests a decline in emotion recognition in the elderly (Ruffman et al., 2008) and other studies reported young-old differences in recognizing complex emotions and mental states in the eyes (Phillips et al., 2002), or in reduced physiological responses to emotion stimuli (Tsai et al., 2000; Kunzmann et al., 2005). However, these trends do not necessarily legitimate to conclude that older people are less emotionally competent than younger people. Their worse performance in emotion recognition tasks might be due to confounding variables such as the nature of the experimental tasks, which are often characterized for presenting non-spontaneous facial expression of emotions and may be highly demanding in the cognitive load required. Moreover, as suggested by Ruffman et al. (2008), older adults might process emotional information not necessarily worse, but just differently than younger adults. In this line a study by Tessitore et al. (2005) suggested that elderly subjects might engage a more distributed neocortical network during the perceptual processing of emotional facial expressions, thus implementing compensatory responses and/or alternative strategies in processing emotions, as the elderly appear to engage cognitive/linguistic systems (e.g., Cabeza et al., 1997; Gunning-Dixon et al., 2003) in the context of a decrease in amygdala activity (e.g., Iidaka et al., 2002; Gunning-Dixon et al., 2003).

### Relationships Between Creativity, Psychometric Intelligence, and Emotional Intelligence

As previously mentioned, creativity has been studied both in relation to general cognitive ability and EI. Creativity seems to represent the intersection where general cognitive ability and EI meet each other.

Many studies have investigated the relationship between psychometric intelligence and creativity. It is known that many processes involved in the development of creativity actually represent skills included in the traditional assessment of cognitive functioning. For example, problem solving, analogic thought, working memory, sustained attention, cognitive flexibility, temporal organization, planning, and evaluation of information adequacy, would appear to be the main cognitive activities activated when searching for an original idea (Dietrich, 2004), and most of them refer to the role of the prefrontal cortex (Carlsson et al., 2000; Abraham et al., 2012). It has been suggested that intelligence predicts creativity especially when it is tested through DT tasks, which explicitly require the subject to be creative at that specific time, in order to evaluate fluency and originality. In contrast, using self-report measures, correlations are weaker with cognitive variables and stronger with other variables, such as personality (Batey et al., 2010). These findings have made it possible to better highlight the contradictory relationship between intelligence and creativity: cognitive performance tasks and DT ability evidently cannot alone explain the wide variety of creative performances (Batey and Furnham, 2006). The relationship between intelligence and creativity is seen in terms of the threshold hypothesis (Jauk et al., 2013). The basic idea behind the threshold hypothesis is that to reach high levels of creativity, high or at least above-average intelligence is required. Guilford (1981) first suggested the existence of a non-linear relationship between the two constructs, with the presence of a high correlation between IQ and creativity scores for an IQ below 120, and a low correlation for an IQ above the average. Guilford explained this discrepancy by referring to the way in which individuals differ in the use of divergent or convergent thinking for problem solving tasks. Subjects with an IQ below 120 would use most of their divergent abilities to arrive at a plausible solution, while those with a higher IQ would rely more on their stable convergent thinking skills. Recent evidence (Jauk et al., 2013) suggested that the threshold hypothesis might vary according to the specific domain of creativity investigated. The threshold of 120 seems to be valid when the ideational originality is more demanding, whereas an IQ of 100 was found to be sufficient for less demanding tasks and an IQ of 85 was found to fit the threshold for a simply quantitative measure of creative potential (i.e., ideational fluency). On the contrary, no threshold was found for creative achievement, i.e., creative achievement benefits from higher intelligence even at fairly high levels of intellectual ability. Finally, intelligence seems to be necessary but not sufficient for creativity and for this reason the need arises to consider also non-cognitive variables, especially emotional and personality aspects (Batey and Furnham, 2006). Recently, Karwowski et al. (2016), in eight studies using different measures of intelligence (i.e., Raven Matrices – Raven, 2000; the Baddeley’s Grammatical Reasoning Test – Baddeley, 1968; Hunt, 2010) and creativity (i.e., Test of Creative Thinking–Drawing Production/Fluency Production/Originality Production – Urban and Jellen, 1996; Test of Creative Imagery Abilities – Jankowska and Karwowski, 2015) observed a consistent pattern that supports the necessary-but-not-sufficient relationship between these two constructs. The authors concluded that although evidence concerning the threshold hypothesis on the creativity–intelligence relationship is mixed, the “necessary condition hypothesis” is clearly corroborated by the results of appropriate tests (see also Karwowski et al., 2017).

In this regard, some studies addressed the issue of the relation between EI, also referred to as EC, and creativity. One line of research investigated the relation between psychopathology and creativity, with contrasting results. Some authors (Andreasen, 1997) found that creative writers and their relatives reported higher percentages of mood disorders compared to controls. Others found not consistent relations between mood disorders and creativity (Waddell, 1998). In this line, Smith and van der Meer (1994) found that patients with psychosomatic disorders who scored higher on standardized measures of creativity produced a greater range of emotional responses, while those with a more restricted pattern of emotional expression scored lower on creativity measures. A study on healthy undergraduate students reported that self-report measures of creativity (specifically, emotional creativity) were not directly related to creative behavior (Ivcevic et al., 2007) but Sánchez-Ruiz et al. (2011) found that EI related to creativity. Possibly EI might be a trait that enhances creativity under specific circumstances, for example when creative activities require the management of emotion (such as in performing) or, alternatively, EI might counterbalance mood disorders in improving creative production (Guastello et al., 2004) because people who possess this skill are more likely to understand their psychological difficulties and direct them into something positive.

In summary, a number of evidences seem to suggest that creativity correlates with both psychometric intelligence and EC but, to the best of our knowledge, no study has directly combined the differential contribution of age, gender, general intelligence, and EC on creativity. Moreover, educational level is considered one of the most influent mediators of lifespan trajectories in the variables considered, thus we included it as a possible predictor of creativity scores.

The aim of the present study was therefore to investigate predictors of creativity, both in terms of style and achievement. In particular, we examined the differential impact of socio-demographic aspects, such as age, gender, and education, and that of psychometric intelligence and EI.

Based on previous literature we expected a significant impact of age and psychometric intelligence on both creativity measures, with a negative relationship concerning age and a positive one for IQ. We also expected EI to further contribute to creativity, over and above intelligence and age. Moreover, we expected gender to have a different role in the creativity dimensions examined, with men showing higher achievements and women better creativity styles.

## Materials and Methods

### Participants

The present study was designed in accordance with the ethical principles for human experimentation in the Declaration of Helsinki and with the local ethics committee. All adult participants signed a written and informed consent form before the study began. All parents of children involved in the study gave their written and informed consent. None of the participants had a history of neurological or psychiatric disease, which was confirmed during an informal interview carried out before the test phase. We recruited 376 participants aged 12–88 (aged *M* = 30.28 years, *SD* = 19.09 years) constituted by 152 males and 224 females (age males, *M* = 29.6 years, *SD* = 19.32 years; age females, *M* = 30.7 years, *SD* = 18.83 years). All participants had normal or corrected-to-normal vision. The sample was selected based on demographic characteristics (educational level) of the Italian population given by ISTAT^1^.

### Materials

#### Creativity Style Questionnaire Revised (CSQ-R; Kumar et al., 1997)

The questionnaire was developed to measure beliefs and strategies regarding creativity. Participants responded to each question on a 5-point Likert scale (ranging from 1 = strongly agree to 5 = strongly disagree). The questionnaire included 78 items falling into seven subscales: belief in unconscious process (e.g., creative ideas occur to me without even thinking about them); use of techniques (e.g., I often let my mind wander to come up with new ideas), use of other people (e.g., I am at my creative best when I work alone); final product orientation (e.g., I usually have a lot of workable ideas); environmental control (e.g., I typically have background music when I am engaged in creative work); superstition (e.g., I have a favorite amulet or piece of clothing that I wear when I am engaged in creative work); and use of sense (e.g., I tend to use my sense of touch in my creative work). Cronbach’s alpha estimated for the seven scales ranged from 0.45 to 0.81, with a median of 0.74. We decided to focus on the total score of the CSQ-R, which is given by the sum of each subscale score, in order to have a quantitative total score to be used in regression and correlational analyses.

#### Creative Achievement Questionnaire (CAQ; Carson et al., 2005)

The CAQ is a self-report checklist consisting of 96 items, divided into three parts. Part One lists 13 different areas of talent, including the 10 domains of artistic and scientific creativity, and three additional domains: individual sports, team sports, and entrepreneurial ventures. Part Two lists concrete achievements in the 10 standard domains of artistic and scientific endeavor (visual arts, music, dance, creative writing, architectural design, humor, theater and film, culinary arts, inventions, and scientific inquiry). The participant is asked to place a checkmark next to the items describing his or her accomplishments. Each domain includes eight ranked questions weighted with a score from 0 to 7. Each domain consists of a “no achievement” item with a weight of zero points (“I have no training or recognized talent in this area”), a “training” item with a weight of one point (“I have taken lessons in this area”), and six additional items of ascending achievement (“I have won a national prize in the fields of science or medicine”). Part Two yields a separate domain score for each of the 10 domains of assessed creative achievement as well as a Total Creative score. Part Three consists of three questions asking the participant to indicate how others perceive him or her, relative to creative characteristics. The questionnaire shows a good test–retest reliability (*r* = 0.81) and internal consistency reliability (Cronbach’s alpha = 0.96). We decided to focus only on the total score of the CAQ.

#### The Short Profile of Emotional Competence (S-PEC; Mikolajczak et al., 2014)

The S-PEC is a 20-item tool that measures 10 dimensions, namely, identification of one’s own emotions (Identification-self; e.g., When I am touched by something, I immediately know what I feel); identification of others’ emotions (Identification – others; e.g., I am good at sensing what others are feeling); understanding of own emotions, (Understanding – self; e.g., I do not always understand why I respond in the way I do); understanding of others’ emotions (Understanding – others; e.g., I do not understand why the people around me respond the way they do); expression of own emotions (Expression – self; e.g., I find it difficult to explain my feelings to others even if I want to); listening to others’ emotions (Listening – others; e.g., Other people tend to confide in me about personal issues); regulation of own emotions (Regulation – self; e.g., When I am angry, I find it easy to calm myself down); regulation of others’ emotions (Regulation – others; e.g., When I see someone who is stressed or anxious, I can easily calm them down); use of own emotions (Use – self; e.g., I never base my personal life choices on my emotions); and use of others’ emotions (Use – others; e.g., I can easily get what I want from others). These dimensions have been found to load on two higher-order factors: intra-personal EI and interpersonal EI, forming together a single EI score. Participants responded to each question on a 5-point Likert scale (ranging from 1 = strongly agree to 5 = strongly disagree). The questionnaire showed moderate to strong correlations between each subscale and the global score (from 0.38 to 0.69), moderate to strong correlations between intrapersonal subscales and the intrapersonal factor (from 0.43 to 0.73) and strong correlations between the interpersonal subscales and the interpersonal factor (0.62–0.74). Intrapersonal EC and Interpersonal EC were moderately correlated (*r* = 0.57).

#### Kaufman Brief Intelligence Test, Second Edition (KBIT-2; Kaufman and Kaufman, 2005; Bonifacci and Nori, 2016)

The Kaufman Brief Intelligence Test, Second Edition (KBIT-2) is a brief, individualized test for measuring verbal and nonverbal intelligence in children and adults from the ages of 4 years through 90 years. It has three subtests, two (vocabulary and riddles) are included in the Verbal IQ score and a Matrices subtest constitutes the Nonverbal scale. Raw scores are converted into standardized scores (mean = 100, *SD* = 15). The internal consistency, specifically the split-half reliability, for the Verbal Scale and Composite IQ was high (*M* = 0.91 and 0.93; respectively). The split-half reliability for the Nonverbal Scale decreased to 0.80 s and 0.90 s. The adjusted test-retest reliability for the Verbal Scale was a mean of 0.91, the mean for the Nonverbal Scale was 0.83, and the mean for the Composite IQ was 0.90.

#### Background Information

Participants were asked to fill out a short questionnaire concerning socio-demographic information, including questions on educational level, age and gender. Other questions served to identify exclusionary criteria, such as questions about physical impairments. For educational level we considered the years of education.

### Procedure

Participants were recruited through advertisements in adult education centers and social centers or through local schools across Italy and they agreed to participate in the study voluntarily. Exclusionary criteria were physical or psychological disabilities that would compromise their ability to fill out the battery described above. Participants took part in the experimental session individually, filling in the questionnaire and KBIT-2. The order of tasks presentation was randomized. The experiment lasted approximately 1 h and 30 min.

## Results

The first set of analyses was carried out using the SPSS package (version 21.0; IBM, United States). First, we performed a bivariate correlation of the main variables included in the study. In **Table 1** the Pearson indexes are reported. Regarding gender, we classified male as -1 and female as 1 as suggested by Howitt and Cramer (2011). Descriptives and *t*-test results about gender differences are reported in **Table 2**. All scores, except CAQ, were distributed normally (asymmetry between -2 and +2; Trochim and Donnelly, 2006). CAQ scores were log-transformed and this allowed to have not-skewed data (Bland et al., 2013).

**Table 1:** Correlations between demographic variables (age and educational level), emotional competence (intrapersonal and interpersonal), and creativity (style and achievement).

	Age	Educational level	IQ composite	Intrapersonal EC	Interpersonal EC	CSQ-R score	CAQ score
Age	—	-0.38^**^	0.01	0.02	-0.13^*^	-0.11^*^	-0.20^**^
Educational level	—	—	0.15^**^	0.03	0.19^**^	0.14^**^	0.07
IQ composite	—	—	—	0.02	0.07	-0.00	0.14^**^
Intrapersonal EC	—	—	—	—	0.40^**^	-0.03	0.06
Interpersonal EC	—	—	—	—	—	0.15^**^	0.12^*^
CSQ-R score	—	—	—	—	—	—	.08

*^∗^p < 0.05 and ^∗∗^p < 0.01.*

**Table 2:** Mean and *SD* values for males and females together with *p*-values and effect size (Cohen’s d) referred to gender differences.

	Males		Females				
	Mean	*SD*	Mean	*SD*	*t*(*df* 374)	*p*	Cohen’s *d*
Age	29.68	19.33	30.85	18.87	-0.586	0.55	0.06
Educational level	3.07	1.02	3.07	1.03	0.009	0.99	0.00
IQ composite	103.22	14.6	100.9	14.86	1.574	0.11	0.17
Intrapersonal EC	31.65	5.07	32.58	5.35	-1.682	0.09	0.18
Interpersonal EC	32.22	5.67	33.35	5.69	-1.896	0.06	0.20
CSQ-R score	241.69	23.1	242.5	24.7	-0.321	0.75	0.03
CAQ score	6.79	10.45	4.62	4.17	2.797	<0.05	0.3

Results from the correlation analyses evidenced, in particular, that Interpersonal, but not Intrapersonal, EC was significantly related to both creativity style and achievement. The relationship between the two components of EC was significant, but with medium effect size, suggesting that the two dimensions are not overlapped. Furthermore, IQ was related only to creative achievements but not to EC and creative style.

### Gender Differences

We ran a set of *t*-tests with gender (males and females) as independent variable and educational level, EC (intrapersonal and interpersonal) and creativity (style and achievement) as dependent variables. **Table 2** reports the mean and *SD* values together with *p*-values and effect size (Cohen’s d). The two groups differed only in CAQ scores, with males reaching higher creativity achievements compared to females.

### Concurrent Predictors of CAQ and CSQ-R Scores

A path analysis was applied using MPlus software (Muthén and Muthén, Muthén and Muthén). Path analysis was used to examine the relationships between the CAQ and CSQ-R scores as dependent variables and the potential predictors assessed, which included gender, age, educational level, composite IQ, intrapersonal and interpersonal EC. Multiple indices were used to evaluate model fit: Chi-square, the root mean square error of approximation (RMSEA), the comparative fit index (CFI), the Tucker–Lewis index (TLI), and the standardized root mean squared residual (SRMR). A non-significant Chi-square, and TLI and CFI values equal to or higher than 0.90 indicate an acceptable model fit; RMSEA and SRMR values close to, respectively, 0.06 and 0.08 or lower indicate an acceptable fit (Hu and Bentler, 1999).

The model’s fit indexes were the following: χ^2^(1) = 1.07, *p* = 0.3, RMSEA = 0.014 (90 % CI = 0.00, -0.138); CFI = 0.998; TLI = 0.976; SRMR = 0.008. Considering Hu and Bentler’s (1999) criteria, the model has good fit indices. The proportion of explained variance for CAQ and CSQ-R scores was relatively low (8.5 and 5.1%, respectively).

**FIGURE 1:**
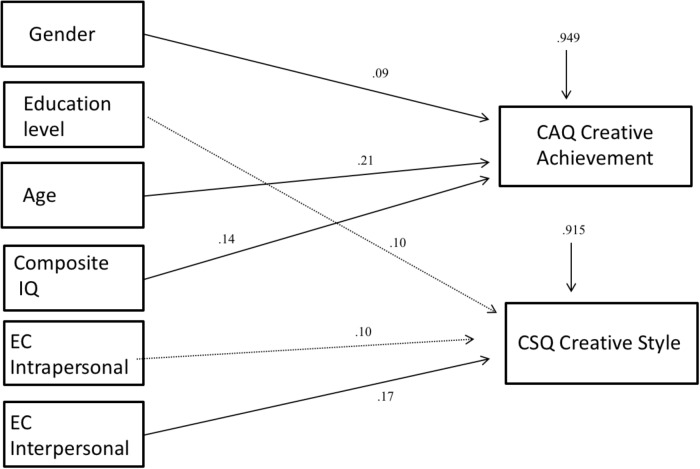
Model predicting CAQ and CSQ scores. Arrows represent significant relationships at *p* < 0.01; missing arrows represent non-signi?cant relationships (*p* > 0.05). The arrows above the CAQ and CSQ squares represent the residual variance for the dependent variables. Fit indices are as follows: RMSEA = 0.014 (90% CI = 0.00, –0.14); CFI = 0.998; TLI = 0.976; and SRMR = 0.008.

**Figure 1** describes the model fitted to the data obtained, and it is possible to observe that CSQ-R was significantly predicted by interpersonal EC and marginally by educational level (*p* = 0.058) and intrapersonal EC (*p* = 0.051). On the other hand, the CAQ score was significantly predicted by gender, age and composite IQ.

## Discussion

The present study focused on an investigation about predictors of creative achievements and creative style, and took into account demographical variables such as age, gender, and educational level together with measures of psychometric intelligence and EC.

First, we explored correlational patterns amongst the considered variables. It emerged that IQ, as expected, was significantly related to educational level, but was not significantly related to EC, considering both intrapersonal and interpersonal domains. This reinforces the evidence that these two constructs are distinct from one another (Mayer et al., 1999). Furthermore, interpersonal and intrapersonal ECs were found to be significantly related, although the correlation index (*r* = 0.40) reflects a medium effect size, suggesting that the two dimensions are not overlapped.

In order to evaluate predictors of creative achievements and creativity style a path analysis was applied.

Considering creative achievements, as measured by the CAQ questionnaire, path analysis showed that age, gender, and Composite IQ were significant predictors of creative achievement. Specifically, younger males, and people with high IQs were found to have higher achievements in creativity, that is, they reported to have reached higher outputs in different areas such as arts, sport, and so on. As far as creative style is concerned, as measured by CSQ-R, it emerged that interpersonal EC is a positive predictor of the measure, whereas IQ and gender did not prove to be significant predictors.

Based on previous literature, these results seem to confirm that gender is related to creative achievements but not to creativity styles (Simonton, 1994; Hardy and Gibson, 2015). In the present study this was also evidenced by the analysis on gender differences, where males were found to have higher achievement scores compared to females, but the two groups did not differ in any other variable considered, including creativity style. In other words, it seems that women have similar creative potential to males but differences emerge in the opportunity to transform this potential into concrete creativity output. Although only speculative, a possible interpretation of this finding might be in relation to the acknowledged gender gap within the labor market (Negrey and Rausch, 2009). This is in line with previous literature (e.g., Baer and Kaufman, 2008) that suggested a limited female accomplishment in creative achievements is possibly due to environmental and/or biological (Abra and Valentine-French, 1991) factors. Future research should extend these findings across different domains.

In the analysis of the role of demographic differences, age proved to be a significant predictor of creative achievements but not of creative style. These data are in line with previous evidence of a decrease in creative achievements in the elderly (Zhang and Niu, 2013), possibly associated with diminished interpersonal competence and cognitive resources, which might limit the opportunities for older people to translate their creative style into concrete creative outputs. Another account of this result regards dedifferentiation in old age (Baltes et al., 1999), which states that in the elderly creativity is mainly influenced by common sources. On the other hand, age did not significantly predict creativity style and this suggests that action should be taken in order to facilitate the expression of creativity in the elderly.

Considering the role of EC, results from the present study suggest that particularly the interpersonal component, and marginally intrapersonal skills, represents a strong predictor of cognitive style but not of creative achievements. This is partially in line with studies that suggest that the openness trait is one of the most consistent personality predictors of creativity (e.g., Silvia et al., 2014; Conner and Silvia, 2015; Karwowski and Lebuda, 2015; Forthmann et al., 2018). In other words, over and above demographic differences in age, gender and education and taking into account cognitive skills connected to intellectual functioning, creative style can be predicted on the basis of people’s skills in recognizing and managing one’s own emotions and those of others. Thus, having good skills in understanding and managing others’ emotions might represent a crucial skill for developing ideas and unconventional solutions. Considering a frequently used model to explain creativity called Knowledge, Skills, Abilities and Other Attributes (the so-called KSAO model), the present study might help in defining some aspects. With regards the “Other” category of the KSAO model, which typically includes personality traits, willingness to take risks (Schmitt and Chan, 1998; Motowidlo, 2003; Stevens, 2012), and motivation (Weitz et al., 1986), our results contribute to better specify the “Other” category by introducing age, gender, interpersonal EC, and Composite IQ. In other words, intelligence could be a necessary but-not-sufficient condition of creativity (e.g., Karwowski et al., 2017).

The design of the present study did not allow to directly test the threshold hypothesis (Guilford, 1981; Jauk et al., 2013) concerning the variation in creativity levels across different IQ levels, but our results suggest that IQ is principally related to creativity achievements and not to creativity style. In accordance with previous literature, the results from the present study suggest that IQ has differential effects on distinct domains of creativity (Jauk et al., 2013). In particular, better achievements seem to require higher IQ, possibly because the fulfillment of creative ideas into concrete outputs requires higher order cognitive skills that encompass the creativity domain.

The generalizability of the present study might be restricted by some limitations. First of all, the data on creativity styles and achievements were collected solely through behavioral questionnaires, so future investigations also including the evaluation of creative potential would be useful. Moreover, although the sample of the study was relatively large, it did not allow for detailed subgroup analysis based on individual differences variables. For example, further research involving a comparison of predictors across different age ranges would be valuable. A final consideration concerns cultural background: the present study was conducted on an Italian population and it would be intriguing to perform cross-cultural studies that considered trajectories for cultural mediated traits such as creativity. Despite these limitations, to the best of our knowledge this is the first study that puts together the analysis of demographic differences, intellectual functioning and EC as predictors of creativity style and achievements.

In summary, the results of the present study seem to be in line with some recent functional evidence, which fosters the idea that creativity arises from a complex interaction between cognitive skills, individual differences variables and EC. Furthermore, the study clearly highlighted a differential influence of cognitive, demographic, and emotional factors on the two distinct domains of creativity considered: achievements and cognitive style. This reinforces the idea that creativity is not an all-in-one construct. Possible implications from the present study could include the implementation of intervention programs that enhance not only the cognitive components associated with creative behavior but also EC and awareness.

## Author Contributions

RN, SS, and PB designed and conceived the study and discussed the results. SS collected data. SS analyzed the data. RN and PB wrote a first draft of the manuscript which has been further revised by all the authors.

## Conflict of Interest Statement

The authors declare that the research was conducted in the absence of any commercial or financial relationships that could be construed as a potential conflict of interest.

## Footnotes

1 http://dati.istat.it/?lang=it

## References

[B1] Abra J. C. Valentine-French S. (1991). Gender differences in creative achievement: a survey of explanations. *Genet. Soc. Gen. Psychol. Monogr.* 117 233–284. 1789886

[B2] Abraham A. Pieritz K. Thybusch K. Rutter B. Kröger S. Schweckendiek J. (2012). Creativity and the brain: uncovering the neural signature of conceptual expansion. *Neuropsychologia* 50 1906–1917. 10.1016/j.neuropsychologia.2012.04.015 22564480

[B3] Adolphs R. (2009). The social brain: neural basis of social knowledge. *Annu. Rev. Psychol.* 60 693–716. 10.1146/annurev.psych.60.110707.163514 18771388 PMC2588649

[B4] Alfonso V. C. Flanagan D. P. Radwan S. (2005). “The impact of the cattell–horn–carroll theory on test development and interpretation of cognitive and academic abilities,” in *Contemporary Intellectual Assessment, Second Edition: Theories, Tests, and Issues*, eds Flanagan D. P. Harrison P. L. (New York, NY: Guilford Publications).

[B5] Amabile T. M. (1983). *The Social Psychology of Creativity.* New York, NY: Springer-Verlag 10.1007/978-1-4612-5533-8

[B6] Anastasi A. (1958). *Differential Psychology: Individual and Group Differences in Behavior.* Oxford: Macmillan.

[B7] Andreasen N. C. (1997). “Creativity and mental illness: prevalence rates in writers and their first-degree relatives,” in *Eminent Creativity, Everyday Creativity, and Health*, eds Runco M. A. Richards R. (Greenwich, CT: Ablex), 7–18. 10.1176/ajp.144.10.1288 3499088

[B8] Baddeley A. D. (1968). A 3 min reasoning test based on grammatical transformation. *Psychon. Sci.* 10 341–342. 10.3758/BF03331551

[B9] Baer J. (1993). *Divergent Thinking and Creativity: A Task-specific Approach.* Hillsdale, NJ: Lawrence Erlbaum Associates, Inc.

[B10] Baer J. Kaufman J. C. (2008). Gender differences in creativity. *J. Creat. Behav.* 42 75–105. 10.1002/j.2162-6057.2008.tb01289.x

[B11] Baltes P. B. Staudinger U. M. Lindenberger U. (1999). Lifespan psychology: theory and application to intellectual functioning. *Annu. Rev. Psychol.* 50 471–507. 10.1146/annurev.psych.50.1.471 15012462

[B12] Batey M. Furnham A. (2006). Creativity, intelligence and personality: a critical review of the scattered literature. *Genet. Soc. Gen. Psychol. Monogr.* 132 355–429. 10.3200/MONO.132.4.355-430 18341234

[B13] Batey M. Furnham A. Saffiulina X. (2010). Intelligence, general knowledge and personality as predictors of creativity. *Learn. Individ. Differ.* 20 532–535. 10.1177/0956797612450883 23184588

[B14] Bland J. M. Altman D. G. Rohlf F. J. (2013). In defence of logarithmic transformations. *Stat. Med.* 32 3766–3768. 10.1002/sim.5772 23983111

[B15] Blum D. Holling H. (2017). Spearman’s law of diminishing returns, a meta-analysis. *Intelligence* 65 60–66. 10.1016/j.intell.2017.07.004

[B16] Bonifacci P. Nori R. (2016). *KBIT-2. Kaufman Brief Intelligence Test Second Edition. Contributo Alla Taratura Italiana [Contribution to Italian Standardization].* Firenze: Giunti-OS.

[B17] Cabello R. Bravo B. N. Latorre J. M. Fernández-Berrocal P. (2014). Ability of university-level education to prevent age-related decline in emotional intelligence. *Front. Aging Neurosci.* 6:37. 10.3389/fnagi.2014.00037 24653697 PMC3949193

[B18] Cabeza R. McIntosh A. R. Tulving E. Nyberg L. Grady C. L. (1997). Age-related differences in effective neural connectivity during encoding and recall. *Neuroreport* 8 3479–3483. 10.1097/00001756-199711100-00013 9427311

[B19] Carlsson I. Wendt P. E. Risberg J. (2000). On the neurobiology of creativity. Differences in frontal activity between high and low creative subjects. *Neuropsychologia* 38 873–885. 10.1016/S0028-3932(99)00128-1 10689061

[B20] Carson S. Peterson J. B. Higgins D. M. (2005). Reliability, validity, and factor structure of the creative achievement questionnaire. *Creat. Res. J.* 17 37–50. 10.1207/s15326934crj1701_4 24597441

[B21] Conner T. S. Silvia P. J. (2015). Creative days: a daily diary study of emotion, personality, and everyday creativity. *Psychol. Aesthet. Creat. Arts* 9 463–470. 10.1037/aca0000022

[B22] Craik F. I. Byrd M. (1982). “Aging and cognitive deficits: the role of attentional resources,” in *Aging and Cognitive Processes*, eds Craik F. I. M. Trehub S. E. (New York, NY: Plenum), 191–211.

[B23] Deary I. J. Penke L. Johnson W. (2010). The neuroscience of human intelligence differences. *Nat. Rev. Neurosci.* 1 201–211. 10.1038/nrn2793 20145623

[B24] Dietrich A. (2004). The cognitive neuroscience of creativity. *Psychon. Bull. Rev.* 11 1011–1026. 10.3758/BF03196731 15875970

[B25] Dudek S. Z. Verreault R. (1989). The creative thinking and ego functioning of children. *Creat. Res. J.* 2 64–86. 10.1080/10400418909534301

[B26] Dunbar K. (1997). “How scientists think: On-line creativity and conceptual change in science,” in *Creative Thought: An Investigation of Conceptual Structures and Processes*, eds Ward T. B. Smith S. M. Vaid J. (Washington, DC: American Psychological Association), 461–493.

[B27] Feist G. J. (1998). A meta-analysis of personality in scientific and artistic creativity. *Pers. Soc. Psychol. Rev.* 2 290–309. 10.1207/s15327957pspr0204_5 15647135

[B28] Finke R. A. Ward T. B. Smith S. M. (1992). *Creative Cognition: Theory, Research, and Applications.* Cambridge, MA: MIT press.

[B29] Fjell A. M. Westlye L. T. Amlien I. Espeseth T. Reinvang I. Raz N. (2009). High consistency of regional cortical thinning in aging across multiple samples. *Cereb. Cortex* 19 2001–2012. 10.1093/cercor/bhn232 19150922 PMC2733683

[B30] Forthmann B. Regehr S. Seidel J. Holling H. Çelik P. Storme M. (2018). Revisiting the interactive effect of multicultural experience and openness to experience on divergent thinking. *Int. J. Intercult. Relat.* 63 135–143. 10.1016/j.ijintrel.2017.10.002

[B31] Getzels J. W. Csikszentmihalyi M. (1976). *The Creative Vision: A Longitudinal Study of Problem Finding in Art.* New York, NY: John Wiley & Sons.

[B32] Guastello S. J. Guastello D. D. Hanson C. A. (2004). Creativity, mood disorders, and emotional intelligence. *J. Creat. Behav.* 38 260–281. 10.1002/j.2162-6057.2004.tb01244.x

[B33] Guilford J. P. (1967). *The Nature of Human Intelligence.* New York, NY: MacGraw-Hill.

[B34] Guilford J. P. (1981). Higher-order structure-of-intellect abilities. *Multivar. Behav. Res.* 16 411–435. 10.1207/s15327906mbr1604_1 26812672

[B35] Gunning-Dixon F. M. Gur R. C. Perkins A. C. Schroeder L. Turner T. Turetsky B. I. (2003). Age-related differences in brain activation during emotional face processing. *Neurobiol. Aging* 24 285–295. 10.1016/S0197-4580(02)00099-4 12498962

[B36] Hardy J. H. Gibson C. (2015). Gender differences in the measurement of creative problem-solving. *J. Creat. Behav.* 51 153–162. 10.1002/jocb.92 29630183

[B37] Hartung J. Doebler P. Schroeders U. Wilhelm O. (2018). Dedifferentiation and differentiation of intelligence in adults across age and years of education. *Intelligence* 69 37–49. 10.1016/j.intell.2018.04.003

[B38] Hildebrandt A. Sommer W. Schacht A. Wilhelm O. (2015). Perceiving and remembering emotional facial expressions—A basic facet of emotional intelligence. *Intelligence* 50 52–67. 10.1016/j.intell.2015.02.003

[B39] Houtz J. C. Ponterotto J. G. Burger C. Marino C. (2010). Problem-solving style and multicultural personality dispositions: a study of construct validity. *Psychol. Rep.* 106 927–938. 10.2466/pr0.106.3.927-938 20712181

[B40] Howitt D. Cramer D. (2011). *Introduction to SPSS Statistics in Psychology: For Version 19 and Earlier.* Upper Saddle River, NJ: Prentice Hall.

[B41] Hu L. T. Bentler P. M. (1999). Cutoff criteria for fit indexes in covariance structure analysis: conventional criteria versus new alternatives. *Struct. Equ. Model.* 6 1–55. 10.1080/10705519909540118

[B42] Hunt E. (2010). *Human Intelligence.* Cambridge, MA: Cambridge University Press 10.1017/CBO9780511781308

[B43] Hutt C. Bhavnani R. (1976). “Predictions from play,” in *Plays Its Role in Development and Evolution*, eds Bruner J. S. Jolly A. Sylva K. (New York, NY: Penguin), 216–219.

[B44] Iidaka T. Okada T. Murata T. Omori M. Kosaka H. Sadato N. (2002). Age-related differences in the medial temporal lobe responses to emotional faces as revealed by fMRI. *Hippocampus* 12 352–362. 10.1002/hipo.1113 12099486

[B45] Ivcevic Z. Brackett M. A. Mayer J. D. (2007). Emotional intelligence and emotional creativity. *J. Pers.* 75 199–235. 10.1111/j.1467-6494.2007.00437.x 17359237

[B46] Jankowska D. M. Karwowski M. (2015). Measuring creative imagery abilities. *Front. Psychol.* 6:1591. 10.3389/fpsyg.2015.01591 26539140 PMC4612655

[B47] Jauk E. Benedek M. Dunst B. Neubauer A. C. (2013). The relationship between intelligence and creativity: new support for threshold hypothesis by means of empirical breakpoint detection. *Intelligence* 41 212–221. 10.1016/j.intell.2013.03.003 23825884 PMC3682183

[B48] Jung R. E. Haier R. J. Yeo R. A. Rowland L. M. Petropoulos H. Levine A. S. (2005). Sex differences in N-acetylaspartate correlates of general intelligence: an 1H-MRS study of normal human brain. *Neuroimage* 26 965–972. 10.1016/j.neuroimage.2005.02.039 15955507

[B49] Jung R. E. Segall J. M. Jeremy Bockholt H. Flores R. A. Smith S. M. Chavez R. S. (2010). Neuroanatomy of creativity. *Hum. Brain Mapp.* 31 398–409. 10.1002/hbm.20874 19722171 PMC2826582

[B50] Karwowski M. Dul J. Gralewski J. Jauk E. Jankowska D. M. Gajda A. (2016). Is creativity without intelligence possible? A necessary condition analysis. *Intelligence* 57 105–117. 10.1016/j.intell.2016.04.006

[B51] Karwowski M. Kaufman J. C. Lebuda I. Szumski G. Firkowska-Mankiewicz A. (2017). Intelligence in childhood and creative achievements in middle-age: the necessary condition approach. *Intelligence* 64 36–44. 10.1016/j.intell.2017.07.001

[B52] Karwowski M. Lebuda I. (2015). The big five, the huge two, and creative self-beliefs: a meta-analysis. *Psychol. Aesthet. Creat. Arts* 10 214–232. 10.1037/aca0000035

[B53] Kaufman A. S. Kaufman N. L. (2005). *Kaufman Brief Intelligence Test* 2nd Edn Circle Pines, MN: American Guidance Service.

[B54] Kaufman J. C. (2015). Why creativity isn’t in IQ tests, why it matters, and why it won’t change anytime soon probably. *J. Intell.* 3 59–72. 10.3390/jintelligence3030059

[B55] Kaufman J. C. (2016). *Creativity 101.* New York, NY: Springer Publishing Company 10.1891/9780826129536

[B56] Kaufman J. C. Beghetto R. A. (2009). Beyond big and little: the four C model of creativity. *Rev. Gen. Psychol.* 13 1–12. 10.1037/a0013688

[B57] Kaufman J. C. Kaufman S. B. Lichtenberger E. O. (2011). Finding creative potential on intelligence tests via divergent production. *Can. J. Sch. Psychol.* 26 83–106. 10.1177/0829573511406511

[B58] Kaufman J. C. Plucker J. A. (2011). “Intelligence and creativity,” in *Cambridge Handbooks in Psychology. The Cambridge Handbook of Intelligence*, eds Sternberg R. J. Kaufman S. B. (New York, NY: Cambridge University Press), 771–783.

[B59] Kaufman S. B. Quilty L. C. Grazioplene R. G. Hirsh J. B. Gray J. R. Peterson J. B. (2016). Openness to experience and intellect differentially predict creative achievement in the arts and sciences. *J. Pers.* 84 248–258. 10.1111/jopy.12156 25487993 PMC4459939

[B60] Kumar V. K. Holman E. R. (1989). *Creativity Styles Questionnaire.* West Chester, PA: Department of Psychology, West Chester University.

[B61] Kumar V. K. Kemmler D. Holman R. (1997). The creativity styles questionnaire revised. *Creat. Res. J.* 10 51–58. 10.1207/s15326934crj1001_6

[B62] Kunzmann U. Kupperbusch C. S. Levenson R. W. (2005). Behavioral inhibition and amplification during emotional arousal: a comparison of two age groups. *Psychol. Aging* 20 144–158. 10.1037/0882-7974.20.1.144 15769220

[B63] Manard M. Carabin D. Jaspar M. Collette F. (2014). Age-related decline in cognitive control: the role of fluid intelligence and processing speed. *BMC Neurosci.* 15:7. 10.1186/1471-2202-15-7 24401034 PMC3890570

[B64] Mathersul D. Palmer D. M. Gur R. C. Gur R. E. Cooper N. Gordon E. (2008). Explicit identification and implicit recognition of facial emotions: II. Core domains and relationships with general cognition. *J. Clin. Exp. Neuropsychol.* 31 278–291. 10.1080/13803390802043619 18720178

[B65] Mayer J. D. Caruso D. L. Salovey P. (1999). Emotional intelligence meets traditional standards for an intelligence. *Intelligence* 27 267–298. 10.1016/S0160-2896(99)00016-1 12934682

[B66] Mayer J. D. Salovey P. (1997). “What is emotional intelligence?,” in *Emotional Development and Emotional Intelligence: Implications for Educators*, eds Salovey P. Sluyter D. (New York, NY: Basic Books), 3–31.

[B67] Mayer J. D. Salovey P. Caruso D. R. (2002). *Mayer–Salovey–Caruso Emotional Intelligence Test (MSCEIT) User Manual.* Toronto: MHS.

[B68] McCann C. Joseph D. L. Newman D. A. Roberts R. D. (2014). Emotional intelligence is a second-stratum factor of intelligence: evidence from hierarchical and bifactor models. *Emotion* 14 358–374. 10.1037/a0034755 24341786

[B69] Mikolajczak M. Brasseur S. Fantini-Hauwel C. (2014). Measuring intrapersonal and interpersonal EQ: the Short Profile of Emotional Competence (S-PEC). *Pers. Individ. Differ.* 65 42–46. 10.1016/j.paid.2014.01.023

[B70] Miller L. J. Myers A. Prinzi L. Mittenberg W. (2009). Changes in intellectual functioning associated with normal aging. *Arch. Clin. Neuropsychol.* 24 681–688. 10.1093/arclin/acp072 19783531

[B71] Motowidlo S. (2003). “Job performance,” in *Handbook of Psychology, Vol. 12 Industrial and Organizational Psychology*, eds Borman W. Ilgen D. Klimoski R. (Hoboken, NJ: John Wiley and Sons), 39–53.

[B72] Muthén L. K. Muthén B. O. (1998–2010). *Mplus User’s Guide* 6th Edn. Los Angeles, CA: Muthén & Muthén.

[B73] Negrey C. Rausch S. D. (2009). Creativity gaps and gender gaps: women, men and place in the United States. *Gend. Place Cult.* 16 517–533. 10.1080/09663690903148408

[B74] Paguio L. P. Hollett N. (1991). Temperament and creativity of preschoolers. *J. Soc. Behav. Pers.* 6 975–982.

[B75] Phillips L. H. MacLean R. D. J. Allen R. (2002). Age and the understanding of emotions: neuropsychological and sociocognitive perspectives. *J. Gerontol. B Psychol. Sci. Soc. Sci.* 57B, 526–530. 10.1093/geronb/57.6.P526 12426435

[B76] Piirto J. (2004). *Understanding Creativity.* Scottsdale, AZ: Great Potential Press.

[B77] Pinker S. Spelke E. (2005). *The science of gender and science: Pinker vs. Spelke, A Debate. Presented at the Mind Brain and Behavior Initiative (MBB).* Cambridge, MA: Harvard University.

[B78] Plucker J. A. Beghetto R. A. Dow G. T. (2004). Why isn’t creativity more important to educational psychologist? Potential, pitfalls and future directions in creativity research. *Educ. Psychol.* 39 83–97. 10.1207/s15326985ep3902_1

[B79] Raven J. (2000). The Raven’s progressive matrices: change and stability over culture and time. *Cogn. Psychol.* 41 1–48. 10.1006/cogp.1999.0735 10945921

[B80] Rhodes M. (1987). “An analysis of creativity,” in *Frontiers of Creativity Research: Beyond the Basics*, ed. Isaksen S. G. (Buffalo, NY: Bearly), 216–222.

[B81] Richardson A. G. (1986). Sex differences in creativity among a sample of Jamaican adolescents. *J. Creat. Behav.* 20:147 10.1002/j.2162-6057.1986.tb00433.x

[B82] Ruffman T. Henry J. D. Livingstone V. Phillips L. H. (2008). A meta-analytic review of emotion recognition and aging: implications for neuropsychological models of aging. *Neurosci. Biobehav. Rev.* 32 863–881. 10.1016/j.neubiorev.2008.01.001 18276008

[B83] Runco M. A. (1991). *Divergent Thinking.* New York, NY: Ablex Publishing.

[B84] Runco M. A. (1994). *Problem Finding, Problem Solving, and Creativity.* Westport, CT: Greenwood Publishing Group.

[B85] Runco M. A. Acar S. (2012). Divergent thinking as an indicator of creative potential. *Creat. Res. J.* 24 66–75. 10.1080/10400419.2012.652929

[B86] Sánchez-Ruiz M. J. Hernández-Torrano D. Pérez-González J. C. Batey M. Petrides K. V. (2011). The relationship between trait emotional intelligence and creativity across subject domains. *Motiv. Emot.* 35 461–473. 10.1007/s11031-011-9227-8

[B87] Sander M. C. Lindenberger U. Werkle-Bergner M. (2012). Lifespan age differences in working memory: a two-component framework. *Neurosci. Biobehav. Rev.* 36 2007–2033. 10.1016/j.neubiorev.2012.06.004 22771333

[B88] Schmidt C. P. Sinor J. (1986). An investigation of the relationships among music audiation, musical creativity, and cognitive style. *J. Res. Music Educ.* 34 160–172. 10.2307/3344746

[B89] Schmitt N. Chan D. (1998). *Personnel Selection: A Theoretical Approach.* Thousand Oaks, CA: SAGE Publications.

[B90] Silvia P. J. Beaty R. E. Nusbaum E. C. (2013). Verbal fluency and creativity: general and specific contributions of broad retrieval ability (Gr) factors to divergent thinking. *Intelligence* 41 328–340. 10.1016/j.intell.2013.05.004

[B91] Silvia P. J. Beaty R. E. Nusbaum E. C. Eddington K. M. Levin-Aspenson H. Kwapil T. R. (2014). Everyday creativity in daily life: an experience-sampling study of “little c” creativity. *Psychol. Aesthet. Creat. Arts* 8 183–188. 10.1037/a0035722

[B92] Simonton D. K. (1994). *Greatness: Who Makes History and Why.* New York, NY: Guilford Press.

[B93] Simonton D. K. (2000). Creativity; cognitive, personal, developmental and social aspects. *Am. Psychol.* 55 151–158. 10.1037/0003-066X.55.1.151 11392859

[B94] Smith G. J. W. Carlsson I. (1983). Creativity in early and middle school years. *Int. J. Behav. Dev.* 6 167–195. 10.1177/016502548300600204

[B95] Smith G. J. W. Carlsson I. (1985). Creativity in middle and late school years. *Int. J. Behav. Dev.* 8 329–343. 10.1177/016502548500800307

[B96] Smith G. J. W. van der Meer G. (1994). “Generative sources of creative functioning,” in *Creativity and Affect*, eds Snow M. P. Runco M. A. (Norwood, NJ: Ablex), 147–167.

[B97] Sternberg R. J. Lubart T. I. (1999). The concept of creativity: prospects and paradigms. *Handb. Creat.* 1 3–15.

[B98] Stevens G. (2012). A critical review of the science and practice of competency modeling. *Hum. Resour. Dev. Rev.* 12 86–107. 10.1177/1534484312456690

[B99] Tessitore A. Hariri A. R. Fera F. Smith W. G. Das S. Weinberger D. R. (2005). Functional changes in the activity of brain regions underlying emotion processing in the elderly. *Psychiatry Res. Neuroimaging* 139 9–18. 10.1016/j.pscychresns.2005.02.009 15936178

[B100] Torrance E. P. Ball O. E. Safter H. T. (1966). *Torrance Tests of Creative Thinking.* Bensenville, IL: Scholastic Testing Service.

[B101] Trochim W. Donnelly J. (2006). *The Research Knowledge Methods Base.* Cincinnati, OH: Atomic Dog Publishing.

[B102] Tsai J. L. Levenson R. W. Carstensen L. L. (2000). Autonomic, subjective, and expressive responses to emotional films in older and younger Chinese Americans and European Americans. *Psychol. Aging* 15 684–693. 10.1037/0882-7974.15.4.684 11144327

[B103] Ueno K. Takahashi T. Takahashi K. Mizukami K. Tanaka Y. Wada Y. (2015). Neurophysiological basis of creativity in healthy elderly people: a multiscale entropy approach. *Clin. Neurophysiol.* 126 524–531. 10.1016/j.clinph.2014.06.032 25066939

[B104] Unsworth N. Spillers G. J. Brewer G. A. (2010). Variation in verbal fluency: a latent variable analysis of clustering, switching, and overall performance. *Q. J. Exp. Psychol.* 64 447–466. 10.1080/17470218.2010.505292 20839136

[B105] Urban K. K. Jellen H. G. (1996). *Manual. Test for Creative Thinking-drawing Production.* Hanover: University of Hanover.

[B106] Vartanian O. Martindale C. Kwiatkowski J. (2003). Creativity and inductive reasoning: the relationship between divergent thinking and performance on wason’s 2—4—6 task. *Q. J. Exp. Psychol. A* 56 1–15. 10.1080/02724980244000567 12745834

[B107] Waddell C. (1998). Creativity and mental illness: is there a link? *Can. J. Psychiatry* 43 166–172. 9533970 10.1177/070674379804300206

[B108] Weisberg R. W. (2006). *Creativity: Understanding Innovation in Problem Solving, Science, Invention, and the Arts.* Hoboken, NJ: John Wiley & Sons.

[B109] Weitz B. A. Sujan H. Sujan M. (1986). Knowledge, motivation, and adaptive behavior: a framework for improving selling effectiveness. *J. Mark.* 50 174–191. 10.2307/1251294

[B110] Wilhelm O. Herzmann G. Kunina O. Danthiir V. Schacht A. Sommer W. (2010). Individual differences in perceiving and recognizing faces—One element of social cognition. *J. Pers. Soc. Psychol.* 99 530. 10.1037/a0019972 20677889

[B111] Wisdom N. M. Mignogna J. Collins R. L. (2012). Variability in wechsler adult intelligence scale-IV subtest performance across age. *Arch. Clin. Neuropsychol.* 27 389–397. 10.1093/arclin/acs041 22512934

[B112] Wu C. H. Cheng Y. Ip H. M. McBride-Chang C. (2005). Age differences in creativity: task structure and knowledge base. *Creat. Res. J.* 17 321–326. 10.1207/s15326934crj1704_3

[B113] Zhang W. Niu W. (2013). Creativity in the later life: factors associated with the creativity of the Chinese elderly. *J. Creat. Behav.* 47 60–76. 10.1002/jocb.23

